# Evaluation of SARS-CoV-2 rapid antigen diagnostic tests for saliva samples

**DOI:** 10.1016/j.heliyon.2022.e08998

**Published:** 2022-02-22

**Authors:** Marie Hagbom, Noelia Carmona-Vicente, Sumit Sharma, Henrik Olsson, Mikael Jämtberg, Åsa Nilsdotter-Augustinsson, Johanna Sjöwall, Johan Nordgren

**Affiliations:** a Division of Molecular Medicine and Virology, Department of Biomedical and Clinical Sciences, Linköping University, 581 85, Linköping, Sweden; b Noviral Sweden AB, Västmannagatan 3, 111 24, Stockholm, Sweden; c Infectious Diseases/Division of Inflammation and Infection, Department of Biomedical and Clinical Sciences, Linköping University, Linköping, Sweden

**Keywords:** SARS-CoV-2, Rapid antigen diagnostic test, Saliva, Infectivity

## Abstract

Using saliva samples would facilitate sample collection, diagnostic feasibility, and mass screening of SARS-CoV-2. We tested two rapid antigen (RAD) immunochromatographic tests designed for detection of SARS-CoV-2 in saliva: Rapid Response™ COVID-19 Antigen Rapid Test Cassette for oral fluids and DIAGNOS™ COVID-19 Antigen Saliva Test. Evaluation of detection limit was performed with purified SARS-CoV-2 nucleocapsid protein and live SARS-CoV-2 virus. Sensitivity and specificity were further evaluated with reverse transcription quantitative PCR (RT-qPCR) positive and negative saliva samples from hospitalized individuals with COVID-19 (n = 39) and healthcare workers (n = 20). DIAGNOS showed higher sensitivity than Rapid Response for both nucleocapsid protein and live virus. The limit of detection of the saliva test from DIAGNOS was further comparable with the Abbott Panbio™ COVID-19 Ag Rapid Test designed for nasopharyngeal samples. DIAGNOS and Rapid Response detected nine (50.0%) and seven (38.9%), respectively, of the 18 RT-qPCR positive saliva samples. All RT-qPCR negative saliva (n = 41) were negative with both tests. Only one of the RT-qPCR positive saliva samples contained infectious virus as determined by cell culture and was also positive using the saliva RADs. The results show that the DIAGNOS may be an important and easy-to-use saliva RAD complement to detect SARS-CoV-2 positive individuals, but validation with a larger sample set is warranted.

## 1 Introduction

There is an urgent need for rapid and easy to use diagnostics for SARS-CoV-2 to limit disease transmission during the ongoing pandemic. The diagnostic gold standard, reverse transcription quantitative PCR (RT-qPCR) have a high specificity and sensitivity, but have limitations with regards to time, cost, and logistics [1, 2].

Several rapid antigen diagnostic tests (RADs) have rapidly been developed for diagnosis of SARS-CoV-2 [3]. At least a few have demonstrated good sensitivity and specificity in comparison to RT-qPCR [3, 4, 5]. Moreover, previous studies have shown that RADs can exhibit high sensitivity in detecting samples containing infectious virus, indicating a high sensitivity to detect contagious individuals [6, 7]. The vast majority of RADs today are designed for nasopharyngeal samples [3, 4, 5]. Using saliva instead of nasopharyngeal swabs has several advantages. It is a noninvasive technique, easy to self-collect with little discomfort, does not require specialized health care personnel and thus reduced risk for the user [8]. Today, there are few RADs designed for saliva available that have been thoroughly validated.

In this study, we have evaluated saliva RADs from two suppliers: Rapid Response™ COVID-19 Antigen Rapid Test Cassette for oral fluids (Rapid Response, BTNX, Markham, Canada) and DIAGNOS™ COVID-19 Antigen Saliva Test (DIAGNOS, Nantong Diagnos Biotechnology, Rugao, China). Both RADs are immunochromatographic assays detecting the SARS-CoV-2 nucleocapsid protein in saliva without any specialized instruments. The tests were first evaluated using SARS-CoV-2 nucleocapsid protein (NCp) and live SARS-CoV-2 virus titrated in saliva or test kit buffer and compared to Abbott Panbio™ COVID-19 Ag Rapid Test (Panbio) designed for nasopharyngeal samples. Finally, the tests were evaluated on saliva samples from individuals hospitalized with COVID-19 and healthcare workers and compared to RT-qPCR. We further correlated the sensitivity of the RADs with regards RT-qPCR Ct-values and infectivity in cell culture.

## 2 Material and methods

To test the sensitivity and detection limit, purified NCp (Nordic Biosite, Sweden, Code: OOEF01087) at a concentration ranging between 500 ng to 5 pg, with 10-fold dilutions was tested with both kits. Each kit buffer was used for making the dilution series. One drop of kit buffer was added, followed by 50 μL of sample containing the protein and two more drops of buffer. Both kits were tested at the same time to make it possible to directly compare the readings. After 15 min of incubation, two persons, independently of each other, made the readings.

Next, saliva which was negative for SARS-CoV-2 were used to make dilutions of cell cultured infectious SARS-CoV-2. A 10-fold dilution series starting from 1:10 to 1:1.000.000, were prepared and used to test both RADs according to the respective manufacturers' instructions. The same saliva without virus addition was used as negative control.

We then tested the RADs on saliva samples obtained from COVID-19 hospitalized individuals (n = 39), collected five to 30 days post symptom onset and from healthcare workers (n = 20) (COVID-19 cohort of Vrinnevi hospital, Norrköping, Sweden). Saliva samples were taken by a nurse between July 2020 and May 2021 and were transported to the laboratory the same day, aliquoted and stored at -80 °C until analysis. Samples were tested simultaneously with both antigen tests to enable a direct comparison. According to instructions, 30 μl of saliva +70 μl of kit buffer were mixed for the Rapid Response, and 40 μl of saliva +35 μl of kit buffer for DIAGNOS. The prepared sample was added to each test stick in the given volume (100 μL and 75 μL, respectively), at room temperature, according to the manufacturer's instructions. After 15 min of incubation, two persons, independently of each other, read all test sticks to determine positivity or negativity. There was no difference in observations made by the two persons and photos were taken for documentation. Viral RNA from the saliva was extracted using QIAmp Viral RNA kit (Qiagen, Hilden, Germany) according to manufactures instructions with the exception that 10 times less sample volume was used, due to need of saliva for both the two RADs as well as cell culture. RNA from the COVID-19 cohort saliva samples (n = 59), RNA from saliva containing titration of SARS-CoV-2 virus and RNA from Vero E6 cultured saliva samples were analyzed using a RT-qPCR for the envelope and RdRp genes. The primers and probes for the envelope gene were E_Sarbeco_P1, E_Sarbeco_F and E_Sarbeco_R from [9]. The primers for RdRp were RdRpF: 5′- GTC ATG TGT GGC GGT TCA CT- ‘3 and RdRpR 5’-AAA CAC TAT TAG CAT AAG CAG TTG-’3, modified from [10], and probe RdRp_Pi from [10]. RT-qPCR was performed on CFX96 (Biorad) using iTaq Universal Probes Supermix (Biorad) with following cycling conditions: reverse transcription at 46 °C for 30 min; followed by initial denaturation at 95 °C for 3 min, followed by 45 cycles of 95 °C for 5 s and 56 °C for 1 min (RdRp primers) or 58 °C for 30 s (envelope primers). All samples were run in duplicates. A sample was considered positive if any of the two RT-qPCR assays were positive. Saliva samples from healthcare workers were only analyzed for the RdRp gene. Confidence intervals for the sensitivity and specificity measurements were calculated with Clopper-Pearson exact method, and confidence intervals for positive and negative predictive values (disease prevalence set at 30.5%; reflecting the ratio of the samples analyzed [18/59]) were calculated using standard logit method [11].

Subsequently, all SARS-CoV-2 PCR positive saliva samples were cultured on Vero E6 cells. Vero E6 cells were cultured in Dulbecco's Modified Eagle Medium (DMEM) supplemented with 10% fetal calf serum (FCS) and gentamycin. Cells were seeded as monolayer in 48-well plates and at time of confluency they were infected with the saliva sample in a biosafety level 3 (BSL3) laboratory, essentially as described [6]. Before infection, the cells were washed two times with DMEM supplemented with 2% FCS and gentamycin. 20 μL of saliva samples were diluted (DMEM supplemented with 2% FCS and gentamycin) to a total volume of 350 μL in a 48 well plate with cells. Samples were blind passaged two times on Vero E6 cells for 3 days each. All cultured saliva samples were tested after passage two for presence of SARS-CoV-2 using the Panbio antigen test and RT-qPCR, to investigate virus replication.

## 3 Results and discussion

**Figure 1:**
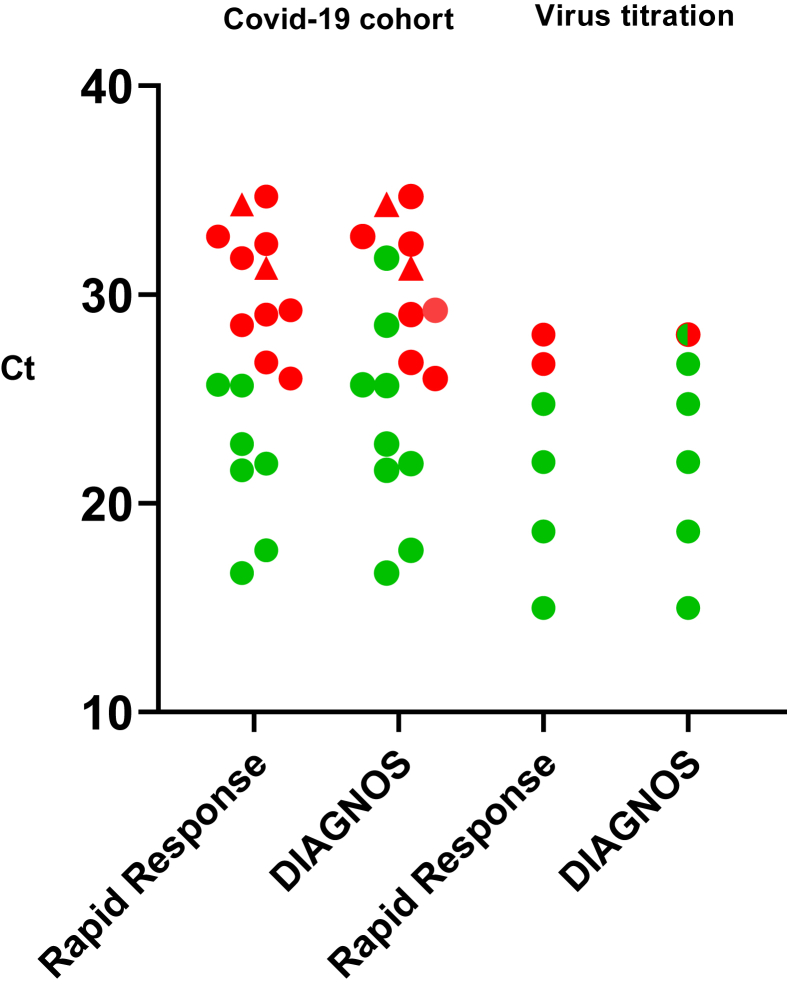
Scatter dot blot indicating positivity (green dot) and negativity (red dot) of the rapid saliva antigen tests in association to RT-qPCR Ct-values for the envelope gene. Two saliva samples were positive only for the RdRp gene (triangle) and are marked with corresponding RdRp Ct-values. DIAGNOS™ detected nine and Rapid Response™ seven of the 18 RT-qPCR positive samples in the COVID-19 cohort (left panel). Using live SARS-CoV-2 titrated in 10-fold dilutions of saliva (right panel), DIAGNOS test exhibited higher sensitivity compared to the Rapid Response test. Green/red dot indicate weak positivity (+/-).

In this study we have evaluated two SARS-CoV-2 RADs designed for use of saliva samples, enabling noninvasive and easy sampling. First, we investigated the detection limit of the assays comparing to Panbio, a widely used RAD for nasopharyngeal samples, using the SARS-CoV-2 NCp protein in a 10-fold concentration range from 500 ng to 5 pg of NCp. The Rapid Response test detected 50 pg, and the DIAGNOS test as well as the Panbio detected 5 pg. These detection limits are in the range of conventional ELISAs [12], which is a standard method of protein detection. We subsequently performed two different sets of virus detection tests, using dilution series of laboratory cultivated SARS-CoV-2 diluted in i) saliva samples, or in ii) test kit buffer, the latter enabling a direct comparison with the Panbio RAD. The DIAGNOS and Rapid Response detected the same virus amount as the Panbio using SARS-CoV-2 diluted in test kit buffer (data not shown), whereas the DIAGNOS test exhibited a lower detection limit for virus diluted in saliva compared to the Rapid Response test (Figure 1).

We subsequently tested the saliva samples collected from patients hospitalized for COVID-19 using the RADs (n = 39, Table 1). The saliva was collected at various time points post symptom onset, with a median of 11 days (range 5–30 days). 18 saliva samples were RT-qPCR positive, and 21 saliva samples were RT-qPCR negative. 16 of the 18 RT-qPCR positive saliva were positive with both envelope and RdRp gene assays, whereas two were only positive with the RdRp assay (Figure 1). In addition to saliva samples of hospitalized patients, saliva samples from health care workers were also included (n = 20), all of which were negative by RT-qPCR. In total, all RT-qPCR negative saliva samples (n = 41), 20 samples from the health care workers and 21 from patients hospitalized with COVID-19, were negative with both tests (specificity 100%; 95% c.i. 91.4–100; Table 2). Of the 18 RT-qPCR positive samples from hospitalized COVID-19 patients, nine (sensitivity 50.0%; 95% c.i. 26.0–74.0) were positive with the DIAGNOS test and seven (sensitivity 38.9%; 95% c.i. 17.3–64.3) with Rapid Response test (Table 2). The positive predictive value (PPV) was 100% for both tests, whereas the negative predictive value (NPV) was 82.0% for DIAGNOS and 78.9% for Rapid Response (Table 2), when disease prevalence was set at 30.5%; reflecting the ratio of the samples analyzed. This is a high prevalence; and within the higher end of the positive rate of COVID-19 tests during the pandemic [13]. The NPV increases as disease prevalence is lower. At a low disease prevalence, high specificity is important to avoid false positives. Both the tests had a 100% specificity, but as the number of negative samples was relatively low (n = 41) in this study, this warrants careful interpretation. As expected, subgroup analysis showed an association between lower Ct-values and RAD positivity (Figure 1). We then proceeded to assess the correlation between infectivity and RT-qPCR results. Only one of the 18 RT-qPCR positive (5.6%) saliva samples showed SARS-CoV-2 replication and associated cytopathic effect (CPE) in Vero E6 cells. This sample was collected 6 days after onset of symptoms and contained a high viral load (Ct 21.6); and was positive with both RADs. Although the numbers are limited, these findings are of interest as they indicate that the saliva RADs may have high sensitivity for detecting contagious individuals, as previously reported for nasopharyngeal RADs [6, 7, 14]. To note, however, three samples with Ct-values of 16.7, 17.7 and 21.9 collected at day 10, 8 and 5 post symptom onset respectively, did not show replication or associated CPE in cell culture. Sample infectivity in saliva, as determined by cell culturing, may not ultimately define whether a person is infectious or not, but detection of infectious virus in cell culture can be a more reliable method than PCR, since PCR does not provide information about virus infectivity [15, 16]. Enzymes and other particles in saliva may also reduce virus infectivity, as well as storage in -80C, thus reducing the sensitivity of the assay in our study, and not accurately reflecting whether the person was infectious at time of sampling.

**Table 1:** Characteristics of the COVID-19 cohort in association to SARS-CoV-2 positivity.

	Saliva samples	Presence of viral RNA in saliva		RAD of RT-qPCR pos saliva		
		RT-qPCR^a^		Rapid Response	DIAGNOS	Infectivity^b^
		Pos	Neg			
COVID-19 hospitalized patients (N)	39	18/39	21/39	7/18 (38.9%)	9/18 (50.0%)	1/18
Gender Male Female	28 (71.8%) 11 (28.2%)	13/28 (46.4%) 5/11 (45.5%)	15/28 (53.6%) 6/11 (54.5%)	5/13 (38.5%) 2/5 (40.0%)	6/13 (46.2%) 3/5 (60.0%)	1/18 0/18
Median age (yr)	57.0 (32–84)	60.0 (39–78)	56.0 (32–84)	59.0 (43–75)	55.0 (43–75)	51
Median DPS	11.0 (5–30)	10.0 (5–30)	11.0 (7–24)	7.0 (5–30)	7.0 (5–30)	6
Healthcare workers	20	0/20	20/20	0/20	0/20	

DPS: Days post symptom onset.

a 16 saliva samples were positive for both the envelope and RdRp genes, whereas 2 samples were only positive for the RdRp gene.

b Containing infectious virus as determined by CPE and replication in cell culture.

**Table 2:** Sensitivity, specificity, positive (PPV) and negative (NPV) predictive values of the rapid saliva antigen tests.

	DIAGNOS (95% CI)	Rapid Response (95% CI)
Sensitivity	50.0% (26.0–74.0)	38.9% (17.3–64.3)
Specificity	100% (91.4–100)	100% (91.4–100)
PPV	100% (NA)	100% (NA)
NPV	82.0% (74.2–87.9)	78.9% (72.1–84.4)

Several studies have evaluated the use of saliva instead of nasopharyngeal swabs as a clinical specimen for COVID-19 diagnostics. Most studies detecting viral RNA in saliva have been evaluated in symptomatic or hospitalized patients, but also from asymptomatic individuals. The results are heterogenous, with saliva showing lower diagnostic accuracy in some studies, while higher in others [8, 14, 17, 18, 19]; with one study reporting a higher concordance to nasopharyngeal samples early after symptom onset [19]. In our study, no major differences were observed for RT-qPCR positivity with regards to days post symptom onset, with positive saliva having a median of 10 and negative saliva a median of 11 days (Table 1). The evaluated saliva RADs, however, were more likely to yield a positive result if the saliva sample was collected early during the infection (median 7 days) (Table 1). Of the RT-qPCR positive saliva samples collected within a week of symptom onset (n = 7), five (71%) and four (57%) were positive with DIAGNOS and Rapid Response, respectively. This was also associated with viral load, with RT-qPCR positive saliva samples collected within one week having a median Ct-value of 25.7 (n = 7) compared to a Ct-value of 29.3 for RT-qPCR samples collected after one week (n = 11). Nevertheless, it is important to consider that onset of symptoms is based on self-reporting and may not be specifically accurate to COVID-19 onset.

This study has other limitations. One major limitation is that no saliva samples were available from onset of symptoms as i) saliva are not routinely used for SARS-CoV-2 diagnostics and ii) patient recruited in the COVID-19 cohort had been ill for several days before seeking hospital care. Samples were taken in a range of 5–30 days, with a median of 11 days post symptom onset and thus do not reflect the intended purpose of the RADs, i.e., testing early after onset of symptoms. Moreover, as we had limited amount of saliva for both the two RADs as well as for the RT-qPCR and cell culture, we had to use less saliva for RNA extraction than is recommended by instructions of the QIAGEN viral extraction kit, thus likely lowering the sensitivity of the RT-qPCR. Finally, a relatively low amount of saliva samples was analyzed, thus warranting careful interpretation of the results.

To conclude, using SARS-CoV-2 NCp protein and titrated live SARS-CoV-2, our results show that the rapid saliva antigen test DIAGNOS had similar limit of detection as Panbio, a widely used RAD developed for nasopharyngeal samples. DIAGNOS further exhibited a 50% sensitivity on RT-qPCR positive saliva from COVID-19 hospitalized patients. Sensitivity was however higher on samples collected early after symptom onset, more in line with the intended use of the RADs, corresponding also to a higher viral load. The number of saliva samples were however small, warranting careful interpretation. The Rapid Response test showed a higher limit of detection as well as lower sensitivity of RT-qPCR positive saliva samples in hospitalized patients compared to DIAGNOS. The overall results suggest that the DIAGNOS saliva antigen test may be a good and easy-to-use complement and possible self-test to be applied for SARS-CoV-2 detection. However, further studies with a larger number of saliva samples collected early during disease onset are needed to validate these saliva tests for large-scale use.

## Ethical statement

Saliva sample collection from COVID-19 patients and health care workers without symptoms were a part of a study approved by the Ethical board in Linköping; Dnr: 2020-02580 and Dnr: 2021-00419. Written informed consent was obtained from all participants in the study.

## Declarations

### Author contribution statement

Marie Hagbom, Noelia Carmona Vicente and Johan Nordgren: Conceived and designed the experiment; Performed the experiments; Analyzed and interpreted the data; Wrote the paper.

Sumit Sharma: Conceived and designed the experiment; Performed the experiments; Wrote the paper.

Henrik Olsson and Mikael Jämtberg: Contributed reagents, materials, analysis tools or data; Wrote the paper.

Åsa Nilsdotter-Augustinsson and Johanna Sjöwall: Analyzed and interpreted the data; Contributed reagents, materials, analysis tools or data; Wrote the paper.

### Funding statement

This study was supported with a coronavirus ALF grant (30320005) from 10.13039/100016670Region Östergötland, Sweden.

### Data availability statement

Data will be made available on request.

### Declaration of interests statement

The authors declare the following conflict of interests: Henrik Olsson and Mikael Jämtberg are Co-chief executive officers at Noviral Sweden AB, which is a distributor of the Rapid Response™ COVID-19 Antigen Rapid Test Cassette and DIAGNOS™ COVID-19 Antigen Saliva Test.

### Additional information

No additional information is available for this paper.
